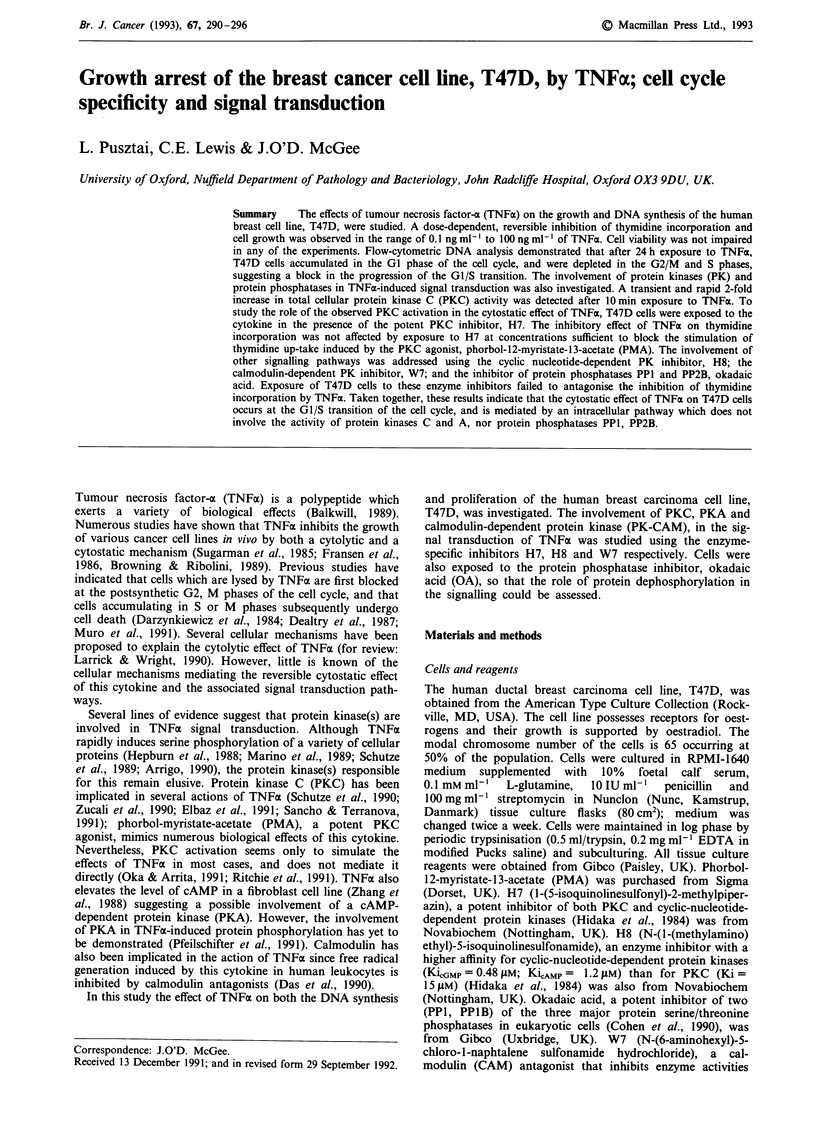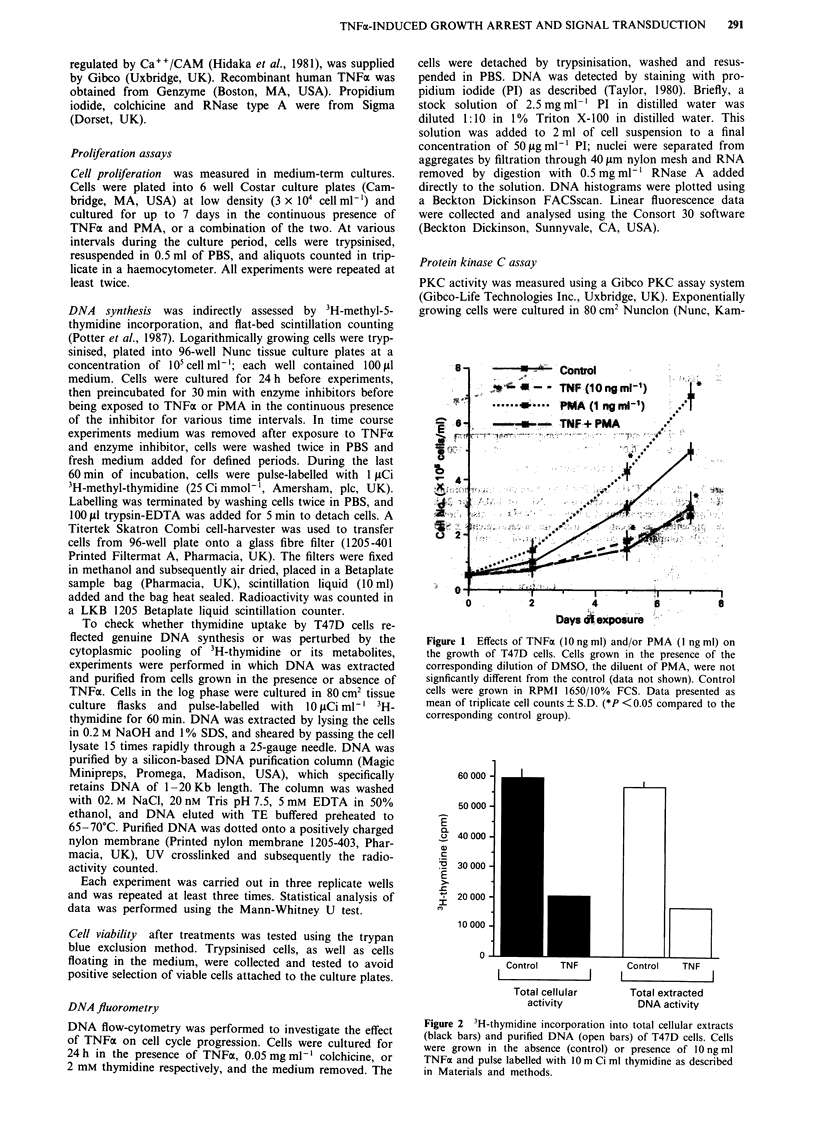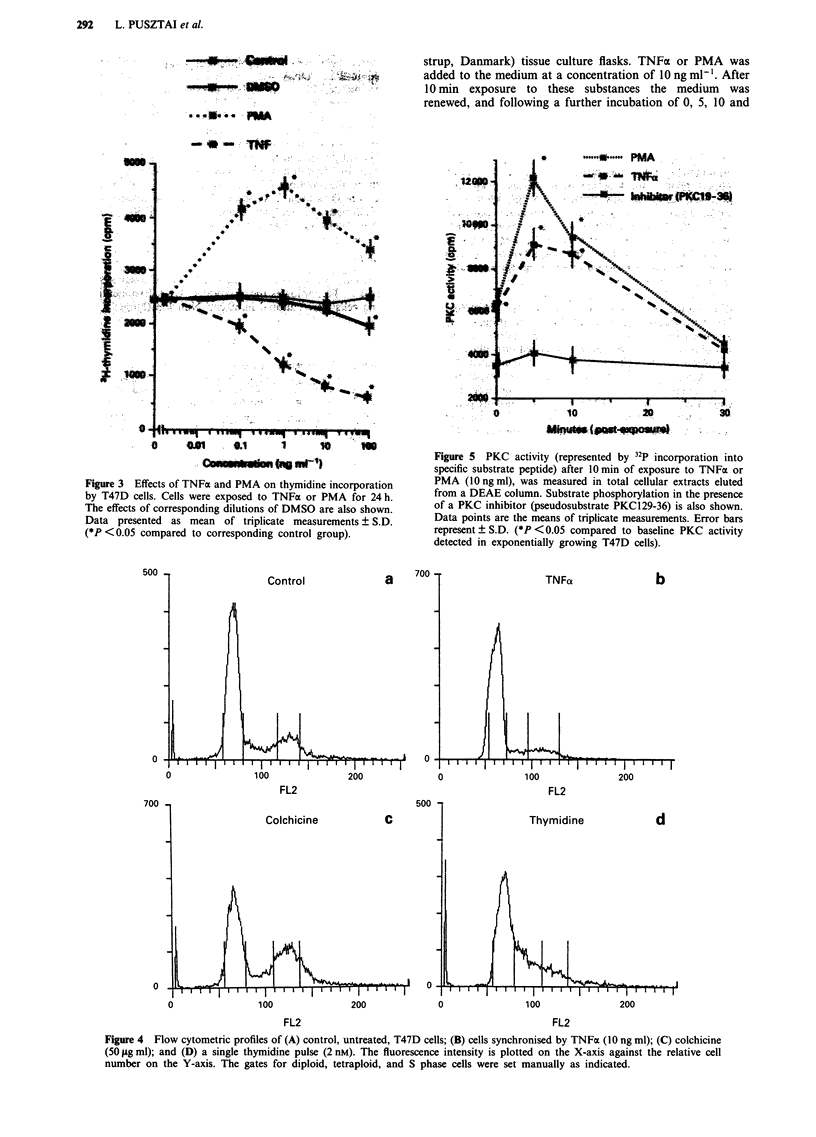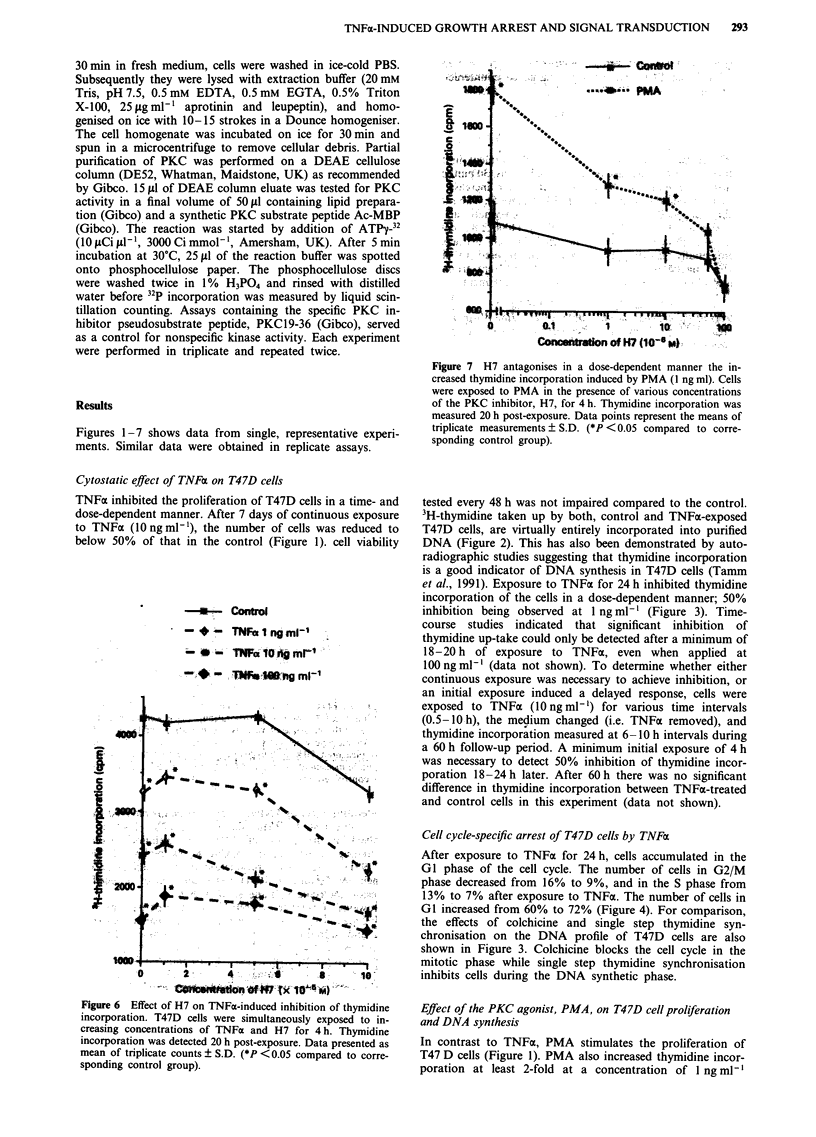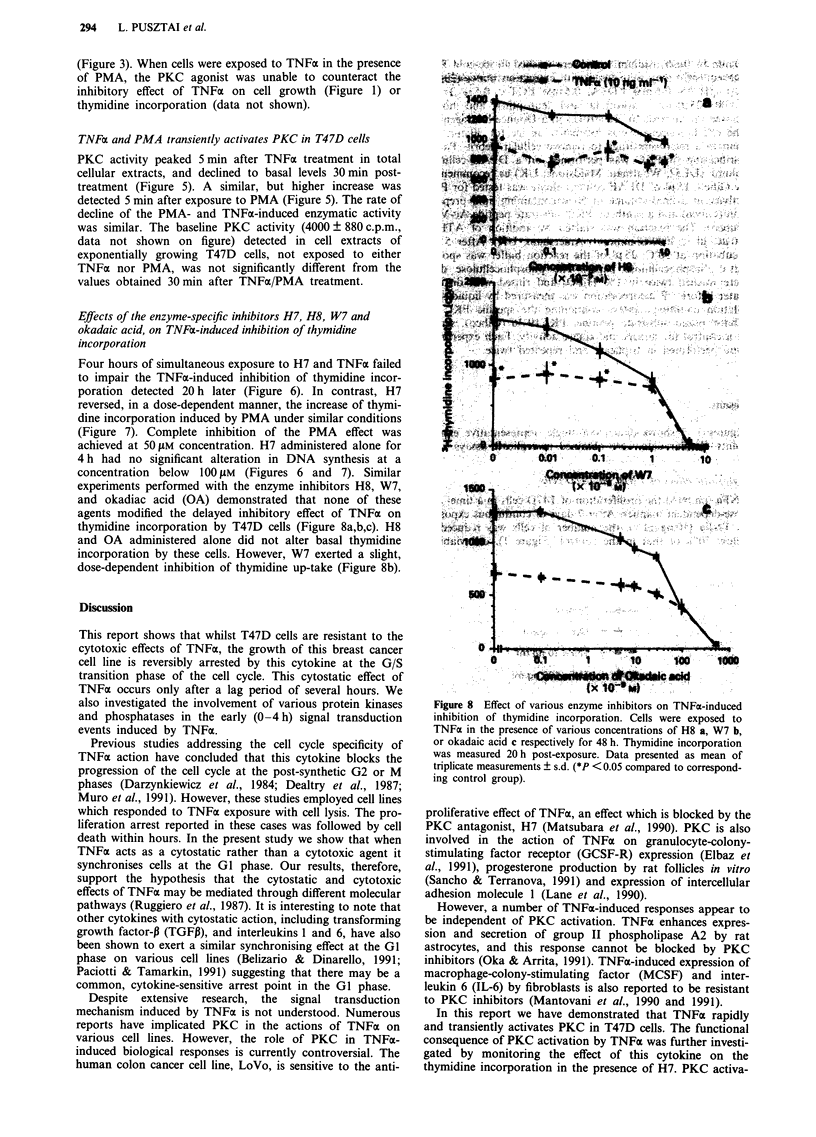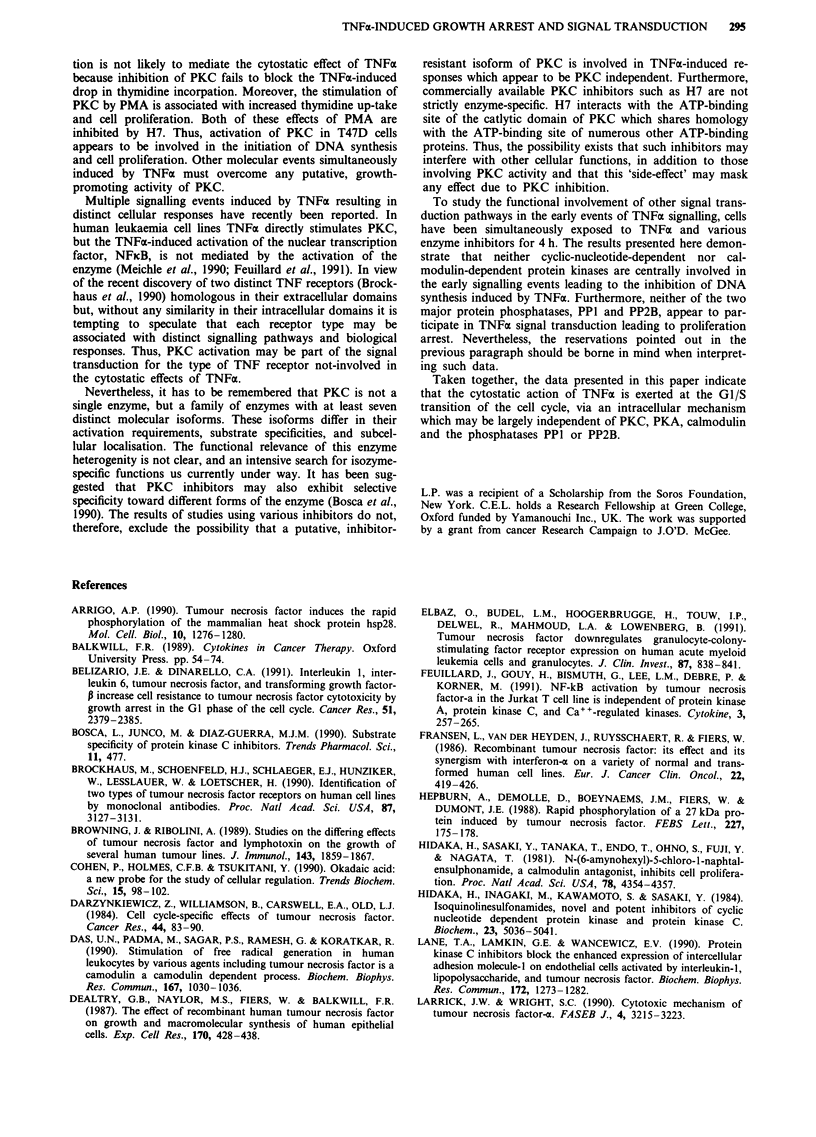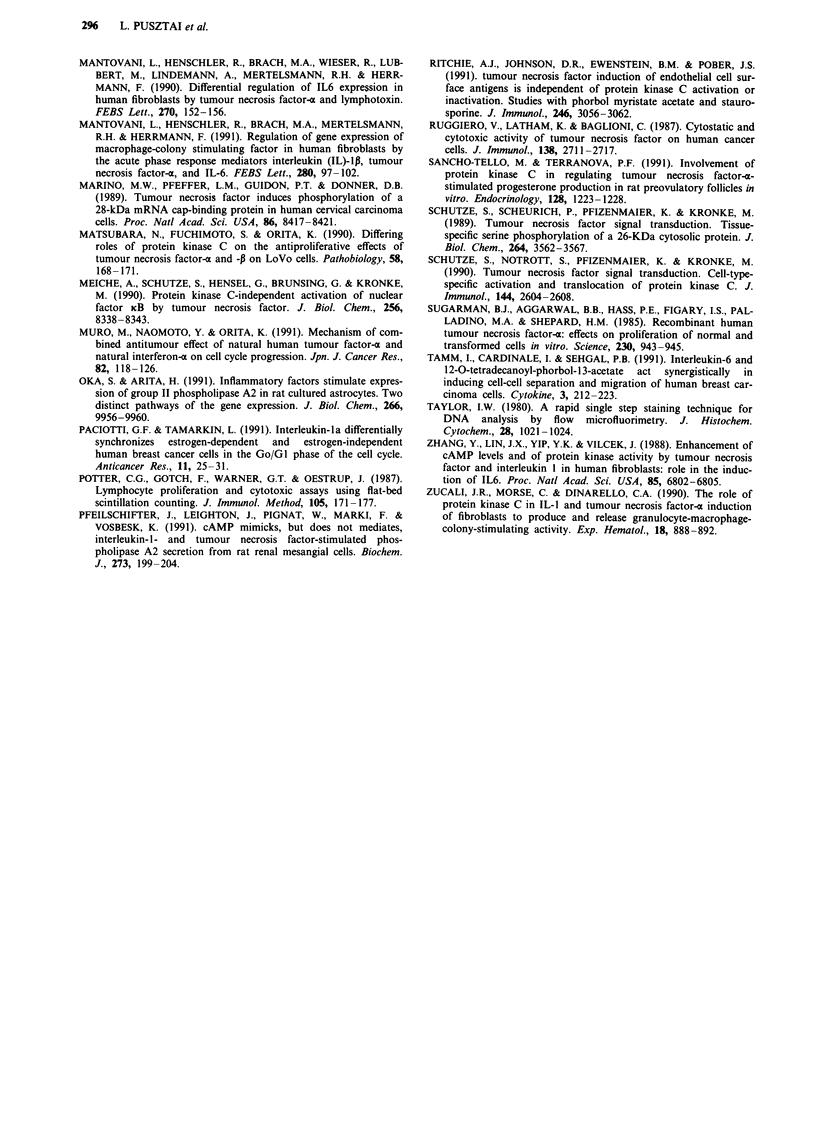# Growth arrest of the breast cancer cell line, T47D, by TNF alpha; cell cycle specificity and signal transduction.

**DOI:** 10.1038/bjc.1993.55

**Published:** 1993-02

**Authors:** L. Pusztai, C. E. Lewis, J. O. McGee

**Affiliations:** University of Oxford, Nuffield Department of Pathology and Bacteriology, John Radcliffe Hospital, UK.

## Abstract

The effects of tumour necrosis factor-alpha (TNF alpha) on the growth and DNA synthesis of the human breast cell line, T47D, were studied. A dose-dependent, reversible inhibition of thymidine incorporation and cell growth was observed in the range of 0.1 ng ml-1 to 100 ng ml-1 of TNF alpha. Cell viability was not impaired in any of the experiments. Flow-cytometric DNA analysis demonstrated that after 24 h exposure to TNF alpha, T47D cells accumulated in the G1 phase of the cell cycle, and were depleted in the G2/M and S phases, suggesting a block in the progression of the G1/S transition. The involvement of protein kinases (PK) and protein phosphatases in TNF alpha-induced signal transduction was also investigated. A transient and rapid 2-fold increase in total cellular protein kinase C (PKC) activity was detected after 10 min exposure to TNF alpha. To study the role of the observed PKC activation in the cytostatic effect of TNF alpha, T47D cells were exposed to the cytokine in the presence of the potent PKC inhibitor, H7. The inhibitory effect of TNF alpha on thymidine incorporation was not affected by exposure to H7 at concentrations sufficient to block the stimulation of thymidine up-take induced by the PKC agonist, phorbol-12-myristate-13-acetate (PMA). The involvement of other signalling pathways was addressed using the cyclic nucleotide-dependent PK inhibitor, H8; the calmodulin-dependent PK inhibitor, W7; and the inhibitor of protein phosphatases PP1 and PP2B, okadaic acid. Exposure of T47D cells to these enzyme inhibitors failed to antagonise the inhibition of thymidine incorporation by TNF alpha. Taken together, these results indicate that the cytostatic effect of TNF alpha on T47D cells occurs at the G1/S transition of the cell cycle, and is mediated by an intracellular pathway which does not involve the activity of protein kinases C and A, nor protein phosphatases PP1, PP2B.


© Macmillan Press Ltd., 1993

### Growth arrest of the breast cancer cell line, T47D, by TNFα; cell cycle specificity and signal transduction

L. Pusztai, C.E. Lewis & J.O'D. McGee

*University of Oxford, Nuffield Department of Pathology and Bacteriology, John Radcliffe Hospital, Oxford OX3 9DU, UK.*

**Summary** The effects of tumour necrosis factor-α (TNFα) on the growth and DNA synthesis of the human breast cell line, T47D, were studied. A dose-dependent, reversible inhibition of thymidine incorporation and cell growth was observed in the range of 0.1 ng ml$^{-1}$ to 100 ng ml$^{-1}$ of TNFα. Cell viability was not impaired in any of the experiments. Flow-cytometric DNA analysis demonstrated that after 24 h exposure to TNFα, T47D cells accumulated in the G1 phase of the cell cycle, and were depleted in the G2/M and S phases, suggesting a block in the progression of the G1/S transition. The involvement of protein kinases (PK) and protein phosphatases in TNFα-induced signal transduction was also investigated. A transient and rapid 2-fold increase in total cellular protein kinase C (PKC) activity was detected after 10 min exposure to TNFα. To study the role of the observed PKC activation in the cytostatic effect of TNFα, T47D cells were exposed to the cytokine in the presence of the potent PKC inhibitor, H7. The inhibitory effect of TNFα on thymidine incorporation was not affected by exposure to H7 at concentrations sufficient to block the stimulation of thymidine up-take induced by the PKC agonist, phorbol-12-myristate-13-acetate (PMA). The involvement of other signalling pathways was addressed using the cyclic nucleotide-dependent PK inhibitor, H8; the calmodulin-dependent PK inhibitor, W7; and the inhibitor of protein phosphatases PP1 and PP2B, okadaic acid. Exposure of T47D cells to these enzyme inhibitors failed to antagonise the inhibition of thymidine incorporation by TNFα. Taken together, these results indicate that the cytostatic effect of TNFα on T47D cells occurs at the G1/S transition of the cell cycle, and is mediated by an intracellular pathway which does not involve the activity of protein kinases C and A, nor protein phosphatases PP1, PP2B.

Tumour necrosis factor-α (TNFα) is a polypeptide which exerts a variety of biological effects (Balkwill, 1989). Numerous studies have shown that TNFα inhibits the growth of various cancer cell lines *in vivo* by both a cytolytic and a cytostatic mechanism (Sugarman *et al*., 1985; Fransen *et al*., 1986, Browning & Ribolini, 1989). Previous studies have indicated that cells which are lysed by TNFα are first blocked at the postsynthetic G2, M phases of the cell cycle, and that cells accumulating in S or M phases subsequently undergo cell death (Darzynkiewicz *et al*., 1984; Dealtry *et al*., 1987; Muro *et al*., 1991). Several cellular mechanisms have been proposed to explain the cytolytic effect of TNFα (for review: Larrick & Wright, 1990). However, little is known of the cellular mechanisms mediating the reversible cytostatic effect of this cytokine and the associated signal transduction pathways.

Several lines of evidence suggest that protein kinase(s) are involved in TNFα signal transduction. Although TNFα rapidly induces serine phosphorylation of a variety of cellular proteins (Hepburn *et al*., 1988; Marino *et al*., 1989; Schutze *et al*., 1989; Arrigo, 1990), the protein kinase(s) responsible for this remain elusive. Protein kinase C (PKC) has been implicated in several actions of TNFα (Schutze *et al*., 1990; Zucali *et al*., 1990; Elbaz *et al*., 1991; Sancho & Terranova, 1991); phorbol-myristate-acetate (PMA), a potent PKC agonist, mimics numerous biological effects of this cytokine. Nevertheless, PKC activation seems only to simulate the effects of TNFα in most cases, and does not mediate it directly (Oka & Arrita, 1991; Ritchie *et al*., 1991). TNFα also elevates the level of cAMP in a fibroblast cell line (Zhang *et al*., 1988) suggesting a possible involvement of a cAMP-dependent protein kinase (PKA). However, the involvement of PKA in TNFα-induced protein phosphorylation has yet to be demonstrated (Pfeilschifter *et al*., 1991). Calmodulin has also been implicated in the action of TNFα since free radical generation induced by this cytokine in human leukocytes is inhibited by calmodulin antagonists (Das *et al*., 1990).

In this study the effect of TNFα on both the DNA synthesis and proliferation of the human breast carcinoma cell line, T47D, was investigated. The involvement of PKC, PKA and calmodulin-dependent protein kinase (PK-CAM), in the signal transduction of TNFα was studied using the enzyme-specific inhibitors H7, H8 and W7 respectively. Cells were also exposed to the protein phosphatase inhibitor, okadaic acid (OA), so that the role of protein dephosphorylation in the signalling could be assessed.

Correspondence: J.O'D. McGee.
Received 13 December 1991; and in revised form 29 September 1992.

### Materials and methods

### *Cells and reagents*

The human ductal breast carcinoma cell line, T47D, was obtained from the American Type Culture Collection (Rockville, MD, USA). The cell line possesses receptors for oestrogens and their growth is supported by oestradiol. The modal chromosome number of the cells is 65 occurring at 50% of the population. Cells were cultured in RPMI-1640 medium supplemented with 10% foetal calf serum, 0.1 mM ml$^{-1}$ L-glutamine, 10 IU ml$^{-1}$ penicillin and 100 mg ml$^{-1}$ streptomycin in Nunclon (Nunc, Kamstrup, Danmark) tissue culture flasks (80 cm$^2$); medium was changed twice a week. Cells were maintained in log phase by periodic trypsinisation (0.5 ml/trypsin, 0.2 mg ml$^{-1}$ EDTA in modified Pucks saline) and subculturing. All tissue culture reagents were obtained from Gibco (Paisley, UK). Phorbol-12-myristate-13-acetate (PMA) was purchased from Sigma (Dorset, UK). H7 (1-(5-isoquinolinesulfonyl)-2-methylpiperazin), a potent inhibitor of both PKC and cyclic-nucleotide-dependent protein kinases (Hidaka *et al*., 1984) was from Novabiochem (Nottingham, UK). H8 (N-(1-(methylamino) ethyl)-5-isoquinolinesulfonamide), an enzyme inhibitor with a higher affinity for cyclic-nucleotide-dependent protein kinases ($Ki_{cGMP}$ = 0.48 μM; $Ki_{cAMP}$ = 1.2 μM) than for PKC (Ki = 15 μM) (Hidaka *et al*., 1984) was also from Novabiochem (Nottingham, UK). Okadaic acid, a potent inhibitor of two (PP1, PP1B) of the three major protein serine/threonine phosphatases in eukaryotic cells (Cohen *et al*., 1990), was from Gibco (Uxbridge, UK). W7 (N-(6-aminohexyl)-5-chloro-1-naphtalene sulfonamide hydrochloride), a calmodulin (CAM) antagonist that inhibits enzyme activities regulated by $Ca^{++}$/CAM (Hidaka *et al.*, 1981), was supplied by Gibco (Uxbridge, UK). Recombinant human TNFα was obtained from Genzyme (Boston, MA, USA). Propidium iodide, colchicine and RNase type A were from Sigma (Dorset, UK).

### *Proliferation assays*

*Cell proliferation* was measured in medium-term cultures. Cells were plated into 6 well Costar culture plates (Cambridge, MA, USA) at low density ($3 \times 10^4$ cell $ml^{-1}$) and cultured for up to 7 days in the continuous presence of TNFα and PMA, or a combination of the two. At various intervals during the culture period, cells were trypsinised, resuspended in 0.5 ml of PBS, and aliquots counted in triplicate in a haemocytometer. All experiments were repeated at least twice.

*DNA synthesis* was indirectly assessed by $^3$H-methyl-5-thymidine incorporation, and flat-bed scintillation counting (Potter *et al.*, 1987). Logarithmically growing cells were trypsinised, plated into 96-well Nunc tissue culture plates at a concentration of $10^5$ cell $ml^{-1}$; each well contained 100 μl medium. Cells were cultured for 24 h before experiments, then preincubated for 30 min with enzyme inhibitors before being exposed to TNFα or PMA in the continuous presence of the inhibitor for various time intervals. In time course experiments medium was removed after exposure to TNFα and enzyme inhibitor, cells were washed twice in PBS and fresh medium added for defined periods. During the last 60 min of incubation, cells were pulse-labelled with 1 μCi $^3$H-methyl-thymidine (25 Ci $mmol^{-1}$, Amersham, plc, UK). Labelling was terminated by washing cells twice in PBS, and 100 μl trypsin-EDTA was added for 5 min to detach cells. A Titertek Skatron Combi cell-harvester was used to transfer cells from 96-well plate onto a glass fibre filter (1205-401 Printed Filtermat A, Pharmacia, UK). The filters were fixed in methanol and subsequently air dried, placed in a Betaplate sample bag (Pharmacia, UK), scintillation liquid (10 ml) added and the bag heat sealed. Radioactivity was counted in a LKB 1205 Betaplate liquid scintillation counter.

To check whether thymidine uptake by T47D cells reflected genuine DNA synthesis or was perturbed by the cytoplasmic pooling of $^3$H-thymidine or its metabolites, experiments were performed in which DNA was extracted and purified from cells grown in the presence or absence of TNFα. Cells in the log phase were cultured in 80 $cm^2$ tissue culture flasks and pulse-labelled with 10 μCi $ml^{-1}$ $^3$H-thymidine for 60 min. DNA was extracted by lysing the cells in 0.2 M NaOH and 1% SDS, and sheared by passing the cell lysate 15 times rapidly through a 25-gauge needle. DNA was purified by a silicon-based DNA purification column (Magic Minipreps, Promega, Madison, USA), which specifically retains DNA of 1–20 Kb length. The column was washed with 02. M NaCl, 20 nM Tris pH 7.5, 5 mM EDTA in 50% ethanol, and DNA eluted with TE buffered preheated to 65–70°C. Purified DNA was dotted onto a positively charged nylon membrane (Printed nylon membrane 1205-403, Pharmacia, UK), UV crosslinked and subsequently the radioactivity counted.

Each experiment was carried out in three replicate wells and was repeated at least three times. Statistical analysis of data was performed using the Mann-Whitney U test.

*Cell viability* after treatments was tested using the trypan blue exclusion method. Trypsinised cells, as well as cells floating in the medium, were collected and tested to avoid positive selection of viable cells attached to the culture plates.

### *DNA fluorometry*

DNA flow-cytometry was performed to investigate the effect of TNFα on cell cycle progression. Cells were cultured for 24 h in the presence of TNFα, 0.05 mg $ml^{-1}$ colchicine, or 2 mM thymidine respectively, and the medium removed. The cells were detached by trypsinisation, washed and resuspended in PBS. DNA was detected by staining with propidium iodide (PI) as described (Taylor, 1980). Briefly, a stock solution of 2.5 mg $ml^{-1}$ PI in distilled water was diluted 1:10 in 1% Triton X-100 in distilled water. This solution was added to 2 ml of cell suspension to a final concentration of 50 μg $ml^{-1}$ PI; nuclei were separated from aggregates by filtration through 40 μm nylon mesh and RNA removed by digestion with 0.5 mg $ml^{-1}$ RNase A added directly to the solution. DNA histograms were plotted using a Beckton Dickinson FACSscan. Linear fluorescence data were collected and analysed using the Consort 30 software (Beckton Dickinson, Sunnyvale, CA, USA).

### *Protein kinase C assay*

PKC activity was measured using a Gibco PKC assay system (Gibco-Life Technologies Inc., Uxbridge, UK). Exponentially growing cells were cultured in 80 $cm^2$ Nunclon (Nunc, Kam-

**Figure 1** Effects of TNFα (10 ng ml) and/or PMA (1 ng ml) on the growth of T47D cells. Cells grown in the presence of the corresponding dilution of DMSO, the diluent of PMA, were not signficantly different from the control (data not shown). Control cells were grown in RPMI 1650/10% FCS. Data presented as mean of triplicate cell counts ± S.D. (*$P$ <0.05 compared to the corresponding control group).

**Figure 2** $^3$H-thymidine incorporation into total cellular extracts (black bars) and purified DNA (open bars) of T47D cells. Cells were grown in the absence (control) or presence of 10 ng ml TNFα and pulse labelled with 10 m Ci ml thymidine as described in Materials and methods.

**Figure 3** Effects of TNFα and PMA on thymidine incorporation by T47D cells. Cells were exposed to TNFα or PMA for 24 h. The effects of corresponding dilutions of DMSO are also shown. Data presented as mean of triplicate measurements ± S.D. (*$P < 0.05$ compared to corresponding control group).

strup, Danmark) tissue culture flasks. TNFα or PMA was added to the medium at a concentration of 10 ng ml$^{-1}$. After 10 min exposure to these substances the medium was renewed, and following a further incubation of 0, 5, 10 and

**Figure 5** PKC activity (represented by $^{32}$P incorporation into specific substrate peptide) after 10 min of exposure to TNFα or PMA (10 ng ml), was measured in total cellular extracts eluted from a DEAE column. Substrate phosphorylation in the presence of a PKC inhibitor (pseudosubstrate PKC129-36) is also shown. Data points are the means of triplicate measurements. Error bars represent ± S.D. (*$P < 0.05$ compared to baseline PKC activity detected in exponentially growing T47D cells).

**Figure 4** Flow cytometric profiles of (**A**) control, untreated, T47D cells; (**B**) cells synchronised by TNFα (10 ng ml); (**C**) colchicine (50 μg ml); and (**D**) a single thymidine pulse (2 nM). The fluorescence intensity is plotted on the X-axis against the relative cell number on the Y-axis. The gates for diploid, tetraploid, and S phase cells were set manually as indicated.

30 min in fresh medium, cells were washed in ice-cold PBS. Subsequently they were lysed with extraction buffer (20 mM Tris, pH 7.5, 0.5 mM EDTA, 0.5 mM EGTA, 0.5% Triton X-100, 25 $\mu g\ ml^{-1}$ aprotinin and leupeptin), and homogenised on ice with 10–15 strokes in a Dounce homogeniser. The cell homogenate was incubated on ice for 30 min and spun in a microcentrifuge to remove cellular debris. Partial purification of PKC was performed on a DEAE cellulose column (DE52, Whatman, Maidstone, UK) as recommended by Gibco. 15 μl of DEAE column eluate was tested for PKC activity in a final volume of 50 μl containing lipid preparation (Gibco) and a synthetic PKC substrate peptide Ac-MBP (Gibco). The reaction was started by addition of $ATP\gamma\text{-}^{32}$ (10 $\mu Ci\ \mu l^{-1}$, 3000 $Ci\ mmol^{-1}$, Amersham, UK). After 5 min incubation at 30°C, 25 μl of the reaction buffer was spotted onto phosphocellulose paper. The phosphocellulose discs were washed twice in 1% $H_3PO_4$ and rinsed with distilled water before $^{32}P$ incorporation was measured by liquid scintillation counting. Assays containing the specific PKC inhibitor pseudosubstrate peptide, PKC19-36 (Gibco), served as a control for nonspecific kinase activity. Each experiment were performed in triplicate and repeated twice.

**Figure 7** H7 antagonises in a dose-dependent manner the increased thymidine incorporation induced by PMA (1 ng ml). Cells were exposed to PMA in the presence of various concentrations of the PKC inhibitor, H7, for 4 h. Thymidine incorporation was measured 20 h post-exposure. Data points represent the means of triplicate measurements ± S.D. (*$P$ < 0.05 compared to corresponding control group).

### Results

Figures 1–7 shows data from single, representative experiments. Similar data were obtained in replicate assays.

### *Cytostatic effect of TNFα on T47D cells*

TNFα inhibited the proliferation of T47D cells in a time- and dose-dependent manner. After 7 days of continuous exposure to TNFα (10 $ng\ ml^{-1}$), the number of cells was reduced to below 50% of that in the control (Figure 1). cell viability tested every 48 h was not impaired compared to the control. $^3$H-thymidine taken up by both, control and TNFα-exposed T47D cells, are virtually entirely incorporated into purified DNA (Figure 2). This has also been demonstrated by autoradiographic studies suggesting that thymidine incorporation is a good indicator of DNA synthesis in T47D cells (Tamm *et al.*, 1991). Exposure to TNFα for 24 h inhibited thymidine incorporation of the cells in a dose-dependent manner; 50% inhibition being observed at 1 $ng\ ml^{-1}$ (Figure 3). Time-course studies indicated that significant inhibition of thymidine up-take could only be detected after a minimum of 18–20 h of exposure to TNFα, even when applied at 100 $ng\ ml^{-1}$ (data not shown). To determine whether either continuous exposure was necessary to achieve inhibition, or an initial exposure induced a delayed response, cells were exposed to TNFα (10 $ng\ ml^{-1}$) for various time intervals (0.5–10 h), the medium changed (i.e. TNFα removed), and thymidine incorporation measured at 6–10 h intervals during a 60 h follow-up period. A minimum initial exposure of 4 h was necessary to detect 50% inhibition of thymidine incorporation 18–24 h later. After 60 h there was no significant difference in thymidine incorporation between TNFα-treated and control cells in this experiment (data not shown).

**Figure 6** Effect of H7 on TNFα-induced inhibition of thymidine incorporation. T47D cells were simultaneously exposed to increasing concentrations of TNFα and H7 for 4 h. Thymidine incorporation was detected 20 h post-exposure. Data presented as mean of triplicate counts ± S.D. (*$P$ < 0.05 compared to corresponding control group).

### *Cell cycle-specific arrest of T47D cells by TNFα*

After exposure to TNFα for 24 h, cells accumulated in the G1 phase of the cell cycle. The number of cells in G2/M phase decreased from 16% to 9%, and in the S phase from 13% to 7% after exposure to TNFα. The number of cells in G1 increased from 60% to 72% (Figure 4). For comparison, the effects of colchicine and single step thymidine synchronisation on the DNA profile of T47D cells are also shown in Figure 3. Colchicine blocks the cell cycle in the mitotic phase while single step thymidine synchronisation inhibits cells during the DNA synthetic phase.

### *Effect of the PKC agonist, PMA, on T47D cell proliferation and DNA synthesis*

In contrast to TNFα, PMA stimulates the proliferation of T47 D cells (Figure 1). PMA also increased thymidine incorporation at least 2-fold at a concentration of 1 $ng\ ml^{-1}$

(Figure 3). When cells were exposed to TNFα in the presence of PMA, the PKC agonist was unable to counteract the inhibitory effect of TNFα on cell growth (Figure 1) or thymidine incorporation (data not shown).

### *TNFα and PMA transiently activates PKC in T47D cells*

PKC activity peaked 5 min after TNFα treatment in total cellular extracts, and declined to basal levels 30 min post-treatment (Figure 5). A similar, but higher increase was detected 5 min after exposure to PMA (Figure 5). The rate of decline of the PMA- and TNFα-induced enzymatic activity was similar. The baseline PKC activity (4000 ± 880 c.p.m., data not shown on figure) detected in cell extracts of exponentially growing T47D cells, not exposed to either TNFα nor PMA, was not significantly different from the values obtained 30 min after TNFα/PMA treatment.

### *Effects of the enzyme-specific inhibitors H7, H8, W7 and okadaic acid, on TNFα-induced inhibition of thymidine incorporation*

Four hours of simultaneous exposure to H7 and TNFα failed to impair the TNFα-induced inhibition of thymidine incorporation detected 20 h later (Figure 6). In contrast, H7 reversed, in a dose-dependent manner, the increase of thymidine incorporation induced by PMA under similar conditions (Figure 7). Complete inhibition of the PMA effect was achieved at 50 μM concentration. H7 administered alone for 4 h had no significant alteration in DNA synthesis at a concentration below 100 μM (Figures 6 and 7). Similar experiments performed with the enzyme inhibitors H8, W7, and okadiac acid (OA) demonstrated that none of these agents modified the delayed inhibitory effect of TNFα on thymidine incorporation by T47D cells (Figure 8a,b,c). H8 and OA administered alone did not alter basal thymidine incorporation by these cells. However, W7 exerted a slight, dose-dependent inhibition of thymidine up-take (Figure 8b).

**Figure 8** Effect of various enzyme inhibitors on TNFα-induced inhibition of thymidine incorporation. Cells were exposed to TNFα in the presence of various concentrations of H8 **a**, W7 **b**, or okadaic acid **c** respectively for 48 h. Thymidine incorporation was measured 20 h post-exposure. Data presented as mean of triplicate measurements ± s.d. (*$P < 0.05$ compared to corresponding control group).

### Discussion

This report shows that whilst T47D cells are resistant to the cytotoxic effects of TNFα, the growth of this breast cancer cell line is reversibly arrested by this cytokine at the G/S transition phase of the cell cycle. This cytostatic effect of TNFα occurs only after a lag period of several hours. We also investigated the involvement of various protein kinases and phosphatases in the early (0–4 h) signal transduction events induced by TNFα.

Previous studies addressing the cell cycle specificity of TNFα action have concluded that this cytokine blocks the progression of the cell cycle at the post-synthetic G2 or M phases (Darzynkiewicz *et al.*, 1984; Dealtry *et al.*, 1987; Muro *et al.*, 1991). However, these studies employed cell lines which responded to TNFα exposure with cell lysis. The proliferation arrest reported in these cases was followed by cell death within hours. In the present study we show that when TNFα acts as a cytostatic rather than a cytotoxic agent it synchronises cells at the G1 phase. Our results, therefore, support the hypothesis that the cytostatic and cytotoxic effects of TNFα may be mediated through different molecular pathways (Ruggiero *et al.*, 1987). It is interesting to note that other cytokines with cytostatic action, including transforming growth factor-β (TGFβ), and interleukins 1 and 6, have also been shown to exert a similar synchronising effect at the G1 phase on various cell lines (Belizario & Dinarello, 1991; Paciotti & Tamarkin, 1991) suggesting that there may be a common, cytokine-sensitive arrest point in the G1 phase.

Despite extensive research, the signal transduction mechanism induced by TNFα is not understood. Numerous reports have implicated PKC in the actions of TNFα on various cell lines. However, the role of PKC in TNFα-induced biological responses is currently controversial. The human colon cancer cell line, LoVo, is sensitive to the anti-proliferative effect of TNFα, an effect which is blocked by the PKC antagonist, H7 (Matsubara *et al.*, 1990). PKC is also involved in the action of TNFα on granulocyte-colony-stimulating factor receptor (GCSF-R) expression (Elbaz *et al.*, 1991), progesterone production by rat follicles *in vitro* (Sancho & Terranova, 1991) and expression of intercellular adhesion molecule 1 (Lane *et al.*, 1990).

However, a number of TNFα-induced responses appear to be independent of PKC activation. TNFα enhances expression and secretion of group II phospholipase A2 by rat astrocytes, and this response cannot be blocked by PKC inhibitors (Oka & Arrita, 1991). TNFα-induced expression of macrophage-colony-stimulating factor (MCSF) and interleukin 6 (IL-6) by fibroblasts is also reported to be resistant to PKC inhibitors (Mantovani *et al.*, 1990 and 1991).

In this report we have demonstrated that TNFα rapidly and transiently activates PKC in T47D cells. The functional consequence of PKC activation by TNFα was further investigated by monitoring the effect of this cytokine on the thymidine incorporation in the presence of H7. PKC activation is not likely to mediate the cytostatic effect of TNFα because inhibition of PKC fails to block the TNFα-induced drop in thymidine incorpation. Moreover, the stimulation of PKC by PMA is associated with increased thymidine up-take and cell proliferation. Both of these effects of PMA are inhibited by H7. Thus, activation of PKC in T47D cells appears to be involved in the initiation of DNA synthesis and cell proliferation. Other molecular events simultaneously induced by TNFα must overcome any putative, growth-promoting activity of PKC.

Multiple signalling events induced by TNFα resulting in distinct cellular responses have recently been reported. In human leukaemia cell lines TNFα directly stimulates PKC, but the TNFα-induced activation of the nuclear transcription factor, NFκB, is not mediated by the activation of the enzyme (Meichle *et al.*, 1990; Feuillard *et al.*, 1991). In view of the recent discovery of two distinct TNF receptors (Brockhaus *et al.*, 1990) homologous in their extracellular domains but, without any similarity in their intracellular domains it is tempting to speculate that each receptor type may be associated with distinct signalling pathways and biological responses. Thus, PKC activation may be part of the signal transduction for the type of TNF receptor not-involved in the cytostatic effects of TNFα.

Nevertheless, it has to be remembered that PKC is not a single enzyme, but a family of enzymes with at least seven distinct molecular isoforms. These isoforms differ in their activation requirements, substrate specificities, and subcellular localisation. The functional relevance of this enzyme heterogenity is not clear, and an intensive search for isozyme-specific functions us currently under way. It has been suggested that PKC inhibitors may also exhibit selective specificity toward different forms of the enzyme (Bosca *et al.*, 1990). The results of studies using various inhibitors do not, therefore, exclude the possibility that a putative, inhibitor-resistant isoform of PKC is involved in TNFα-induced responses which appear to be PKC independent. Furthermore, commercially available PKC inhibitors such as H7 are not strictly enzyme-specific. H7 interacts with the ATP-binding site of the catlytic domain of PKC which shares homology with the ATP-binding site of numerous other ATP-binding proteins. Thus, the possibility exists that such inhibitors may interfere with other cellular functions, in addition to those involving PKC activity and that this 'side-effect' may mask any effect due to PKC inhibition.

To study the functional involvement of other signal transduction pathways in the early events of TNFα signalling, cells have been simultaneously exposed to TNFα and various enzyme inhibitors for 4 h. The results presented here demonstrate that neither cyclic-nucleotide-dependent nor calmodulin-dependent protein kinases are centrally involved in the early signalling events leading to the inhibition of DNA synthesis induced by TNFα. Furthermore, neither of the two major protein phosphatases, PP1 and PP2B, appear to participate in TNFα signal transduction leading to proliferation arrest. Nevertheless, the reservations pointed out in the previous paragraph should be borne in mind when interpreting such data.

Taken together, the data presented in this paper indicate that the cytostatic action of TNFα is exerted at the G1/S transition of the cell cycle, via an intracellular mechanism which may be largely independent of PKC, PKA, calmodulin and the phosphatases PP1 or PP2B.

L.P. was a recipient of a Scholarship from the Soros Foundation, New York. C.E.L. holds a Research Fellowship at Green College, Oxford funded by Yamanouchi Inc., UK. The work was supported by a grant from cancer Research Campaign to J.O'D. McGee.

### References

ARRIGO, A.P. (1990). Tumour necrosis factor induces the rapid phosphorylation of the mammalian heat shock protein hsp28. *Mol. Cell. Biol.*, **10**, 1276–1280.

BALKWILL, F.R. (1989). *Cytokines in Cancer Therapy*. Oxford University Press. pp. 54–74.

BELIZARIO, J.E. & DINARELLO, C.A. (1991). Interleukin 1, interleukin 6, tumour necrosis factor, and transforming growth factor-β increase cell resistance to tumour necrosis factor cytotoxicity by growth arrest in the G1 phase of the cell cycle. *Cancer Res.*, **51**, 2379–2385.

BOSCA, L., JUNCO, M. & DIAZ-GUERRA, M.J.M. (1990). Substrate specificity of protein kinase C inhibitors. *Trends Pharmacol. Sci.*, **11**, 477.

BROCKHAUS, M., SCHOENFELD, H.J., SCHLAEGER, E.J., HUNZIKER, W., LESSLAUER, W. & LOETSCHER, H. (1990). Identification of two types of tumour necrosis factor receptors on human cell lines by monoclonal antibodies. *Proc. Natl Acad. Sci. USA*, **87**, 3127–3131.

BROWNING, J. & RIBOLINI, A. (1989). Studies on the differing effects of tumour necrosis factor and lymphotoxin on the growth of several human tumour lines. *J. Immunol.*, **143**, 1859–1867.

COHEN, P., HOLMES, C.F.B. & TSUKITANI, Y. (1990). Okadaic acid: a new probe for the study of cellular regulation. *Trends Biochem. Sci.*, **15**, 98–102.

DARZYNKIEWICZ, Z., WILLIAMSON, B., CARSWELL, E.A., OLD, L.J. (1984). Cell cycle-specific effects of tumour necrosis factor. *Cancer Res.*, **44**, 83–90.

DAS, U.N., PADMA, M., SAGAR, P.S., RAMESH, G. & KORATKAR, R. (1990). Stimulation of free radical generation in human leukocytes by various agents including tumour necrosis factor is a camodulin a camodulin dependent process. *Biochem. Biophys. Res. Commun.*, **167**, 1030–1036.

DEALTRY, G.B., NAYLOR, M.S., FIERS, W. & BALKWILL, F.R. (1987). The effect of recombinant human tumour necrosis factor on growth and macromolecular synthesis of human epithelial cells. *Exp. Cell Res.*, **170**, 428–438.

ELBAZ, O., BUDEL, L.M., HOOGERBRUGGE, H., TOUW, I.P., DELWEL, R., MAHMOUD, L.A. & LOWENBERG, B. (1991). Tumour necrosis factor downregulates granulocyte-colony-stimulating factor receptor expression on human acute myeloid leukemia cells and granulocytes. *J. Clin. Invest.*, **87**, 838–841.

FEUILLARD, J., GOUY, H., BISMUTH, G., LEE, L.M., DEBRE, P. & KORNER, M. (1991). NF-kB activation by tumour necrosis factor-a in the Jurkat T cell line is independent of protein kinase A, protein kinase C, and $Ca^{++}$-regulated kinases. *Cytokine*, **3**, 257–265.

FRANSEN, L., VAN DER HEYDEN, J., RUYSSCHAERT, R. & FIERS, W. (1986). Recombinant tumour necrosis factor: its effect and its synergism with interferon-α on a variety of normal and transformed human cell lines. *Eur. J. Cancer Clin. Oncol.*, **22**, 419–426.

HEPBURN, A., DEMOLLE, D., BOEYNAEMS, J.M., FIERS, W. & DUMONT, J.E. (1988). Rapid phosphorylation of a 27 kDa protein induced by tumour necrosis factor. *FEBS Lett.*, **227**, 175–178.

HIDAKA, H., SASAKI, Y., TANAKA, T., ENDO, T., OHNO, S., FUJI, Y. & NAGATA, T. (1981). N-(6-amynohexyl)-5-chloro-1-naphtalensulphonamide, a calmodulin antagonist, inhibits cell proliferation. *Proc. Natl Acad. Sci. USA*, **78**, 4354–4357.

HIDAKA, H., INAGAKI, M., KAWAMOTO, S. & SASAKI, Y. (1984). Isoquinolinesulfonamides, novel and potent inhibitors of cyclic nucleotide dependent protein kinase and protein kinase C. *Biochem.*, **23**, 5036–5041.

LANE, T.A., LAMKIN, G.E. & WANCEWICZ, E.V. (1990). Protein kinase C inhibitors block the enhanced expression of intercellular adhesion molecule-1 on endothelial cells activated by interleukin-1, lipopolysaccharide, and tumour necrosis factor. *Biochem. Biophys. Res. Commun.*, **172**, 1273–1282.

LARRICK, J.W. & WRIGHT, S.C. (1990). Cytotoxic mechanism of tumour necrosis factor-α. *FASEB J.*, **4**, 3215–3223.

MANTOVANI, L., HENSCHLER, R., BRACH, M.A., WIESER, R., LUBBERT, M., LINDEMANN, A., MERTELSMANN, R.H. & HERRMANN, F. (1990). Differential regulation of IL6 expression in human fibroblasts by tumour necrosis factor-α and lymphotoxin. *FEBS Lett.*, **270**, 152-156.

MANTOVANI, L., HENSCHLER, R., BRACH, M.A., MERTELSMANN, R.H. & HERRMANN, F. (1991). Regulation of gene expression of macrophage-colony stimulating factor in human fibroblasts by the acute phase response mediators interleukin (IL)-1β, tumour necrosis factor-α, and IL-6. *FEBS Lett.*, **280**, 97-102.

MARINO, M.W., PFEFFER, L.M., GUIDON, P.T. & DONNER, D.B. (1989). Tumour necrosis factor induces phosphorylation of a 28-kDa mRNA cap-binding protein in human cervical carcinoma cells. *Proc. Natl Acad. Sci. USA*, **86**, 8417-8421.

MATSUBARA, N., FUCHIMOTO, S. & ORITA, K. (1990). Differing roles of protein kinase C on the antiproliferative effects of tumour necrosis factor-α and -β on LoVo cells. *Pathobiology*, **58**, 168-171.

MEICHE, A., SCHUTZE, S., HENSEL, G., BRUNSING, G. & KRONKE, M. (1990). Protein kinase C-independent activation of nuclear factor κB by tumour necrosis factor. *J. Biol. Chem.*, **256**, 8338-8343.

MURO, M., NAOMOTO, Y. & ORITA, K. (1991). Mechanism of combined antitumour effect of natural human tumour factor-α and natural interferon-α on cell cycle progression. *Jpn. J. Cancer Res.*, **82**, 118-126.

OKA, S. & ARITA, H. (1991). Inflammatory factors stimulate expression of group II phospholipase A2 in rat cultured astrocytes. Two distinct pathways of the gene expression. *J. Biol. Chem.*, **266**, 9956-9960.

PACIOTTI, G.F. & TAMARKIN, L. (1991). Interleukin-1a differentially synchronizes estrogen-dependent and estrogen-independent human breast cancer cells in the Go/G1 phase of the cell cycle. *Anticancer Res.*, **11**, 25-31.

POTTER, C.G., GOTCH, F., WARNER, G.T. & OESTRUP, J. (1987). Lymphocyte proliferation and cytotoxic assays using flat-bed scintillation counting. *J. Immunol. Method*, **105**, 171-177.

PFEILSCHIFTER, J., LEIGHTON, J., PIGNAT, W., MARKI, F. & VOSBESK, K. (1991). cAMP mimicks, but does not mediates, interleukin-1- and tumour necrosis factor-stimulated phospholipase A2 secretion from rat renal mesangial cells. *Biochem. J.*, **273**, 199-204.

RITCHIE, A.J., JOHNSON, D.R., EWENSTEIN, B.M. & POBER, J.S. (1991). tumour necrosis factor induction of endothelial cell surface antigens is independent of protein kinase C activation or inactivation. Studies with phorbol myristate acetate and staurosporine. *J. Immunol.*, **246**, 3056-3062.

RUGGIERO, V., LATHAM, K. & BAGLIONI, C. (1987). Cytostatic and cytotoxic activity of tumour necrosis factor on human cancer cells. *J. Immunol.*, **138**, 2711-2717.

SANCHO-TELLO, M. & TERRANOVA, P.F. (1991). Involvement of protein kinase C in regulating tumour necrosis factor-α-stimulated progesterone production in rat preovulatory follicles *in vitro*. *Endocrinology*, **128**, 1223-1228.

SCHUTZE, S., SCHEURICH, P., PFIZENMAIER, K. & KRONKE, M. (1989). Tumour necrosis factor signal transduction. Tissue-specific serine phosphorylation of a 26-KDa cytosolic protein. *J. Biol. Chem.*, **264**, 3562-3567.

SCHUTZE, S., NOTROTT, S., PFIZENMAIER, K. & KRONKE, M. (1990). Tumour necrosis factor signal transduction. Cell-type-specific activation and translocation of protein kinase C. *J. Immunol.*, **144**, 2604-2608.

SUGARMAN, B.J., AGGARWAL, B.B., HASS, P.E., FIGARY, I.S., PALLADINO, M.A. & SHEPARD, H.M. (1985). Recombinant human tumour necrosis factor-α: effects on proliferation of normal and transformed cells *in vitro*. *Science*, **230**, 943-945.

TAMM, I., CARDINALE, I. & SEHGAL, P.B. (1991). Interleukin-6 and 12-O-tetradecanoyl-phorbol-13-acetate act synergistically in inducing cell-cell separation and migration of human breast carcinoma cells. *Cytokine*, **3**, 212-223.

TAYLOR, I.W. (1980). A rapid single step staining technique for DNA analysis by flow microfluorimetry. *J. Histochem. Cytochem.*, **28**, 1021-1024.

ZHANG, Y., LIN, J.X., YIP, Y.K. & VILCEK, J. (1988). Enhancement of cAMP levels and of protein kinase activity by tumour necrosis factor and interleukin 1 in human fibroblasts: role in the induction of IL6. *Proc. Natl Acad. Sci. USA*, **85**, 6802-6805.

ZUCALI, J.R., MORSE, C. & DINARELLO, C.A. (1990). The role of protein kinase C in IL-1 and tumour necrosis factor-α induction of fibroblasts to produce and release granulocyte-macrophage-colony-stimulating activity. *Exp. Hematol.*, **18**, 888-892.